# Nanoconfinement-Enhanced CO_2_ Retention in Fenamate-Loaded Silica Aerogels

**DOI:** 10.3390/molecules31183248

**Published:** 2026-09-14

**Authors:** Konstantin Belov, Maria Ikim, Varvara Demina, Valentina Sobornova, Maria Mochalova, Natalia Menshutina, Michael Kiselev, Leonid Trakhtenberg, Ilya Khodov

**Affiliations:** 1 G.A. Krestov Institute of Solution Chemistry of the Russian Academy of Sciences, Ivanovo 153045, Russia; 2 N.N. Semenov Federal Research Center for Chemical Physics RAS, Moscow 119991, Russiatrakh@chph.ras.ru (L.T.); 3 Department of Chemical and Pharmaceutical Engineering, D. Mendeleev University of Chemical Technology of Russia, Moscow 125047, Russia; 4 Moscow Center for Advanced Studies, Moscow 123592, Russia

**Keywords:** mefenamic acid, flufenamic acid, aerogels, carbon dioxide, temperature-programmed desorption/oxidation, BET analysis

## Abstract

The interaction between carbon dioxide and molecularly confined pharmaceutical compounds can result in CO_2_ retention that exceeds that achieved through conventional physical adsorption. This study investigates CO_2_ retention following sorption in hydrophilic and hydrophobic silica aerogels containing the fenamates mefenamic acid and flufenamic acid. The Thermal stability of the retained CO_2_ was characterized using temperature-programmed oxidation and temperature-programmed desorption measurements. Concurrently, single-point nitrogen adsorption measurements monitored relative changes in the apparent accessible surface area of the porous matrix. Untreated silica aerogels did not exhibit a significant CO_2_ desorption peak at elevated temperatures. In contrast, all composites containing fenamates exhibited an additional high-temperature desorption step beginning at approximately 225 °C, indicating enhanced CO_2_ retention after sorption once the external CO_2_ layer was removed. This characteristic persisted following preliminary thermal treatment, suggesting it is not solely attributable to residual volatile substances. Surface-area measurements indicated minimal changes in the original aerogels after the CO_2_ cycle, whereas composites with flufenamic acid demonstrated more pronounced alterations. Considering previous nuclear magnetic resonance, spectroscopic, and computational studies, these findings suggest a combined effect of nanoconfinement, surface-dependent interfacial phenomena, and specific interactions involving the fenamate-containing phase. Reversible chemical interactions may contribute to the observed retention, although the current measurements do not allow for quantitative separation of their effects from those of physical confinement.

## 1. Introduction

Fenamates, including mefenamic acid (MFA) and flufenamic acid (FFA), are conformationally flexible nonsteroidal anti-inflammatory drugs whose molecular and solid-state structures exhibit high sensitivity to their local environment [1,2,3,4,5]. This pronounced sensitivity renders fenamates effective molecular probes for examining how solvent interactions, spatial confinement, and intermolecular forces influence the relationship between molecular conformation and solid-state organization [1,2,6,7,8]. Supercritical carbon dioxide (scCO_2_) is particularly advantageous in this context because its solvent properties can be continuously adjusted by varying pressure and temperature, facilitating both molecular processing and crystallization control without reliance on conventional organic solvents [9,10,11]. Systematic investigations of the solubility of various fenamate derivatives in scCO_2_ have revealed a strong dependence on molecular structure and thermodynamic parameters [9,10,12].

In addition to influencing solubility, scCO_2_ can modify the molecular organization of fenamates [13,14]. Nuclear magnetic resonance (NMR) studies of MFA and FFA have demonstrated alterations in their conformational states within CO_2_-containing media, thereby linking the molecular environment to the formation and stability of distinct solid forms [13]. More broadly, the significance of spatial confinement has been established for FFA, where nanoconfinement alters the pathways of polymorphic transformation compared to the bulk material [3,15,16]. Collectively, these findings indicate that the structural behavior of fenamates is determined not only by their intrinsic molecular properties but also by the nanoscale environment in which they are situated.

Porous aerogels offer a highly versatile platform for environmental control due to their combination of high surface area, interconnected porosity, and chemically tunable internal surfaces [17,18,19]. The application of scCO_2_ for loading pharmaceutical compounds into aerogels has consequently received significant attention [20,21,22,23,24,25,26]. Nonsteroidal anti-inflammatory drugs such as nimesulide, ketoprofen, and diclofenac have been successfully incorporated into biopolymer aerogels using scCO_2_ [26]. Conversely, studies involving ibuprofen and ketoprofen in mesoporous silica have shown that drug loading is influenced by both solubility in scCO_2_ and interactions with the porous matrix [17,27,28,29]. Notably, the quantity of drug retained after depressurization does not necessarily reflect the equilibrium adsorbed amount, as precipitation during depressurization can substantially affect the measured loading [17].

In the case of fenamates, the porous matrix is increasingly regarded as an active component of the molecular environment rather than merely a passive carrier [30,31]. Incorporation of MFA into silica aerogels has been shown to alter their conformational equilibrium through interactions with the silica surface, while FFA-containing silica aerogels display modifications in both the drug’s molecular environment and the composite’s sorption properties [31,32,33,34]. Studies involving cellulose-based aerogels further indicate that the chemical composition of the matrix can dictate the localization and structural state of confined MFA and FFA. Collectively, these results establish a coupled fenamate–surface–CO_2_ system in which molecular confinement, surface interactions, and the CO_2_ environment must be considered as interdependent factors.

A significant consequence of this coupling has received relatively limited attention: the state and persistence of CO_2_ following removal of the external CO_2_ phase [35,36,37,38]. Most previous studies have focused on CO_2_ uptake, diffusion, and molecular localization while the porous material is in contact with CO_2_-containing media [39,40,41]. These measurements yield insights into the dynamic equilibrium established during exposure, but do not directly determine whether a portion of CO_2_ remains strongly retained after the external CO_2_ reservoir is removed. This distinction is especially relevant for fenamate-containing aerogels, as interactions with the organic component may generate CO_2_ states distinct from those present on pristine silica surfaces.

This question is further informed by recent molecular-level investigations of fenamate–CO_2_ interactions [31,42,43]. Previous work has demonstrated that MFA reacts with CO_2_ in scCO_2_ and, notably, that confinement within an aerogel can modify the preferred reaction pathway and decrease the temperature at which reaction products are detectable [42]. Similar studies of FFA have confirmed its chemical reactivity with CO_2_ and the formation of a distinct reaction product [43]. These findings indicate that molecular interactions within the confined fenamate phase may have implications extending beyond the reaction itself, potentially affecting the persistence of CO_2_ within the porous composite after removal of the external CO_2_ phase [9,44].

A key unresolved issue is whether the enhanced post-sorption retention observed in fenamate-containing aerogels results primarily from physical confinement of CO_2_, specific intermolecular interactions, chemical binding, or a combination of these mechanisms. Clarifying these contributions is important because CO_2_ localized within a confined nanoporous environment may display markedly different retention behavior compared to CO_2_ undergoing conventional reversible physisorption on a bare silica surface. This study therefore investigates the thermal persistence of retained CO_2_ by employing comparative TPO/TPD measurements of pristine and fenamate-containing aerogels to determine whether the incorporation of the fenamate phase introduces an additional CO_2_-retention mechanism.

The present study examines the post-sorption retention of CO_2_ in MFA- and FFA-containing silica aerogels using temperature-programmed oxidation/desorption (TPO/TPD) in conjunction with single-point Brunauer–Emmett–Teller (BET) measurements of specific surface area [45]. This methodology allows for assessment of the thermal persistence of retained CO_2_ as well as changes in the textural properties of the porous matrix [46,47,48]. Pristine hydrophilic and hydrophobic silica aerogels, despite possessing high specific surface areas, do not display a significant high-temperature CO_2_-release feature. In contrast, both fenamate-containing composites exhibit an additional high-temperature CO_2_-release process [47,49]. The persistence of this feature after calcination, along with the relatively minor changes in the textural characteristics of the pristine aerogels following the CO_2_ cycle, indicates that alterations in the silica pore structure alone cannot fully explain the observed CO_2_ retention.

These findings highlight a key characteristic of fenamate–aerogel systems: the incorporation of a molecular dopant can produce a population of CO_2_ species that remains strongly retained after removal of the external CO_2_ phase [38]. Together with analysis of prior NMR, spectroscopic, and computational evidence for specific fenamate–surface and fenamate–CO_2_ interactions, the current results support a model in which CO_2_ retention is governed by the combined effects of nanoconfinement, interfacial interactions, and specific interactions involving the fenamate-containing phase [50]. Therefore, the most relevant descriptor for these materials is not solely their CO_2_ uptake under pressure, but also the molecular state, thermal stability, and persistence of CO_2_ following removal of the external reservoir.

## 2. Results and Discussion

### 2.1. TPO/TPD Characterization of Pristine and Fenamate-Doped Silica Aerogels

**Figure 1:**
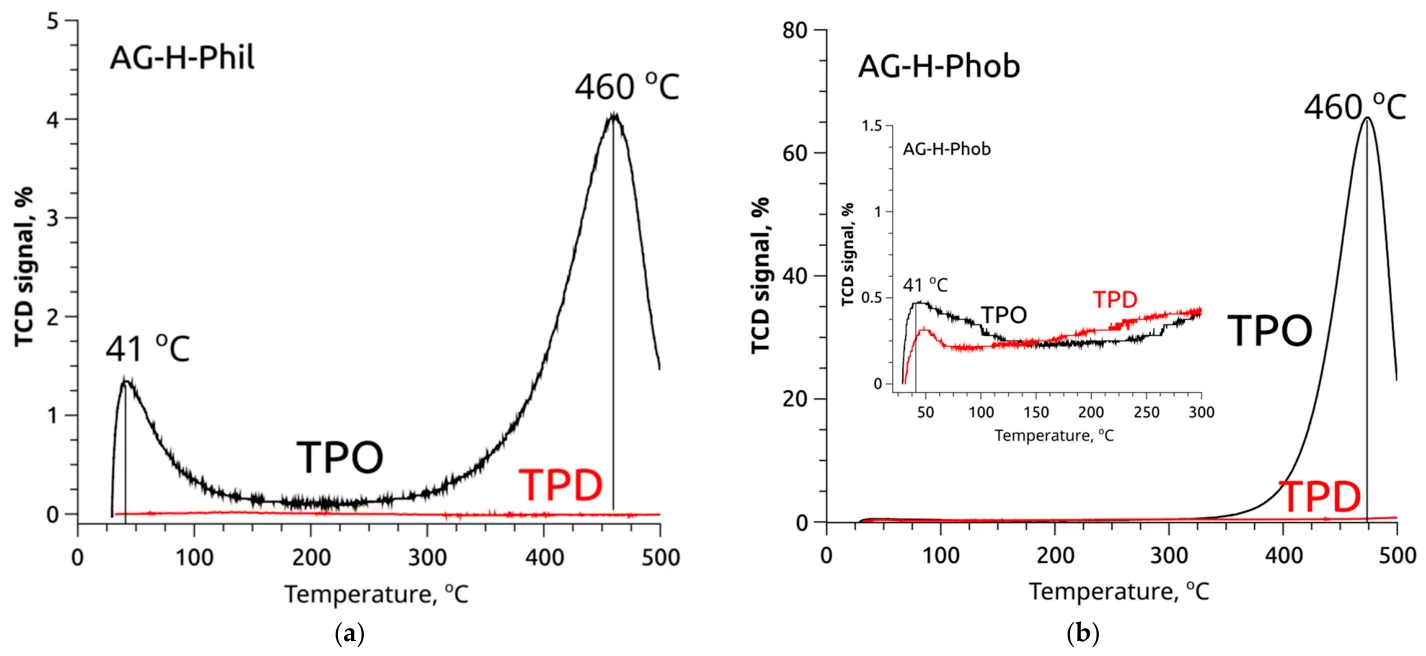
TPO (black) and TPD (red) curves for AG-H-Phil (**a**) and AG-H-Phob (**b**) recorded over the temperature range from 30 °C to 500 °C.

Initially, the TPO/TPD behavior of the hydrophilic (H-Phil) and hydrophobic (H-Phob) aerogel (AG)-based materials was compared (Figure 1). Establishing the baseline behavior of pristine, undoped materials is essential for interpreting the TPO and TPD profiles of the fenamate-loaded composites.

The AG-H-Phil sample exhibits a pronounced peak at approximately 41 °C; however, a signal in the same temperature region is also present for AG-H-Phob. In both materials, the TCD response appears immediately after the experiment commences. This low-temperature signal is most likely associated with the removal of physically adsorbed water, residual solvent from aerogel synthesis, and other volatile species within the pore structure. A high-temperature signal centered at approximately 460 °C is observed in both materials, but with markedly different intensities: for AG-H-Phob, the TCD response reaches approximately 66%, whereas for AG-H-Phil it is only about 4%. Previous IR spectroscopic studies have shown that hydrophobic silica aerogels are characterized predominantly by Si–CH_3_ groups at the pore surface, whereas hydrophilic materials contain Si–OH functionalities [51,52,53]. The pronounced high-temperature response of AG-H-Phob may therefore be attributed to the oxidation of surface CH_3_ groups and their conversion into more hydrophilic surface functionalities, as previously reported [54,55,56]. Neither material exhibits a pronounced TPD peak, indicating that the pristine aerogel matrices do not retain CO_2_ for extended periods under the applied flow-through conditions. These baseline observations provide a reference for assessing the behavior of the fenamate-doped composites, specifically mefenamic (MFA) and flufenamic (FFA) acid-based composites AG-H-Phil+MFA, AG-H-Phob+MFA, AG-H-Phil+FFA, and AG-H-Phob+FFA.

**Figure 2:**
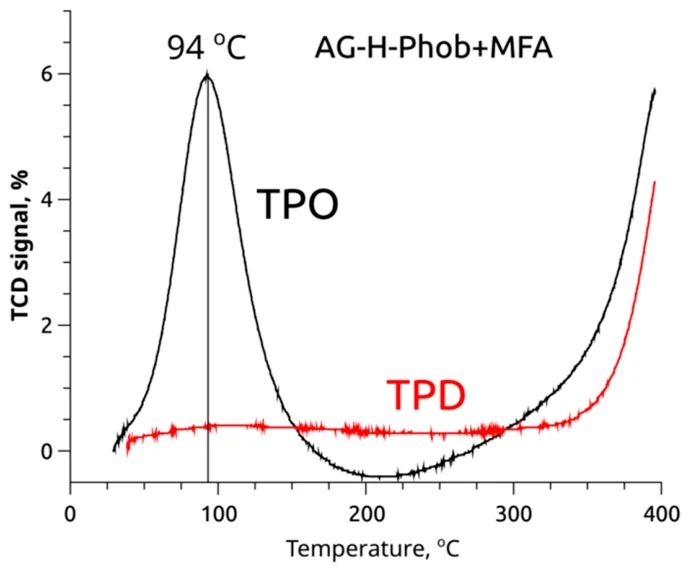
TPO (black) and TPD (red) curves for AG-H-Phob+MFA recorded over the temperature range from 30 °C to 400 °C.

The initial composite investigated was AG-H-Phob+MFA (Figure 2). To identify characteristic patterns of CO_2_ interaction with both the composite matrix and the dopant, the upper temperature limit was reduced to 400 °C to minimize thermal degradation of mefenamic acid.

The first peak is shifted to a higher temperature, approximately 94 °C, and becomes more intense. This behavior may result from additional contributions of residual solvents, particularly isopropanol (Tb = 82.4 °C), used during sample preparation. Changes in the TCD response may also reflect partial removal of fenamate located near the external surface of the composite. At higher temperatures, the TPO profile exhibits a shoulder corresponding to the feature previously observed for the undoped matrices, but with substantially increased intensity. The most significant difference is observed in the TPD profile, where a relatively symmetric high-temperature signal appears, which was absent in pristine AG-H-Phob. This feature indicates that CO_2_ is retained in the composite structure considerably longer than in the pristine aerogel and suggests the presence of additional interactions between CO_2_ and the fenamate-containing structure.

This observation is consistent with previously reported NMR results for nanocrystalline cellulose aerogel-based composites exposed to supercritical CO_2_ [31]. In those systems, the correlation times (τ_c_) of the composite materials (0.38 and 0.25 h) exceeded those of the corresponding pristine support matrices (0.15 h), indicating stronger retention of CO_2_ within the composite structure. The present TPD results provide complementary evidence for this behavior under flow-through conditions. While the NMR measurements were conducted in a cell with continuous scCO_2_ as the surrounding medium, TPD monitors the release of previously retained CO_2_ during a temperature ramp. The emergence of a high-temperature TPD signal indicates that the composite retains CO_2_ even after the external supply ceases.

These findings also clarify results reported in a previous study of silica-aerogel systems containing the same dopants [32]. In that work, substantially longer correlation times were obtained for the pristine silica matrices (1.2 h), whereas lower values were observed for the MFA- and FFA-containing composites (0.7 and 0.14 h, respectively). This behavior was interpreted in terms of the availability of sites or “vacancies” for CO_2_ localization within the pore structure. The combined results suggest that the different trends observed in the two experimental configurations are attributable to fundamentally different measurement conditions. Under continuous scCO_2_ exposure, the pristine silica aerogel provides numerous accessible sites for CO_2_ localization and can therefore exhibit relatively long correlation times. In contrast, once the external CO_2_ supply is removed, as in the flow-through TPD experiment, the pristine aerogel does not show significant long-term CO_2_ retention. The presence of the fenamate introduces additional interactions that allow a fraction of CO_2_ to remain associated with the composite structure during subsequent heating.

The fact that the pristine aerogels and those containing fenamates behave differently shows that the retention observed cannot be explained merely as conventional reversible physisorption on the silica surface. When the physically trapped CO_2_ in the pristine matrices is released under the flow-through TPD conditions, no sharp high-temperature desorption peak is obtained. By contrast, when MFA or FFA is incorporated, there appears an extra population of CO_2_ which stays bound to the composite down to much higher temperatures. This result is in agreement with there being stronger specific interactions between CO_2_ and the fenamate-containing phase.

It is also possible that a chemical effect is responsible for this retention. Earlier spectroscopic and computational studies have shown that both MFA and FFA are able to enter into chemical interactions with CO_2_ under the right conditions, and that spatial confinement can affect the formation and stability of the products thus obtained. It follows that the high-temperature feature seen in the present composites might be due at least in part to CO_2_ species that are chemically bound or otherwise strongly associated. However, this experiment does not offer a direct molecular identification of the retained species, and it cannot be ruled out that a contribution comes from strongly confined physisorbed CO_2_.

Therefore, silica aerogel without dopant is well suited for rapid CO_2_ uptake and prolonged CO_2_ localization under conditions resembling impregnation, when a continuous CO_2_ supply is maintained. Under flow-through TPO/TPD conditions, however, the retention mechanism is more complex, and the presence of the dopant becomes critical for maintaining CO_2_ within the material after the external CO_2_ supply is discontinued. This distinction reconciles the contrasting NMR and TPD observations and underscores the importance of experimental configuration when interpreting CO_2_ dynamics in porous materials.

A plausible explanation for the enhanced CO_2_ retention in fenamate-containing composites is the presence of chemical interactions between the dopant and CO_2_, indicating that chemisorption may significantly contribute to the observed effects. Such behavior was previously demonstrated for MFA in scCO_2_ and under confinement within a nanocrystalline-cellulose aerogel [42]. Using IR spectroscopy and quantum-chemical calculations, it was shown that the reaction between MFA and CO_2_ occurs in saturated scCO_2_ solution above approximately 190 °C. In contrast, confinement within the aerogel lowers the onset temperature to approximately 100 °C. Similar behavior was subsequently demonstrated for FFA [43]. In that case, the reaction in the bulk solution occurred at approximately 100 °C and around 200 bar, producing an amorphous reaction product that remained stable in air for an extended period.

These previous observations are particularly relevant to the present TPD results because they provide independent evidence that the fenamate molecules are capable of specific chemical interactions with CO_2_. The appearance of the high-temperature CO_2_ desorption feature in the present composites is therefore consistent with a contribution from chemical binding in addition to physical confinement and other intermolecular interactions. In this context, the higher temperature required for CO_2_ release may reflect the greater stability of the retained CO_2_-containing species compared with the weakly and reversibly localized CO_2_ observed in the pristine matrices.

However, the TPD experiment measures the thermal release of CO_2_ and does not directly identify the molecular structure of the retained species. The observed high-temperature feature should therefore not be assigned exclusively to chemisorption. Rather, the present results support a mechanistic model in which physical confinement, specific fenamate–CO_2_ interactions, and a possible reversible chemical component jointly contribute to the enhanced post-sorption CO_2_ retention.

**Figure 3:**
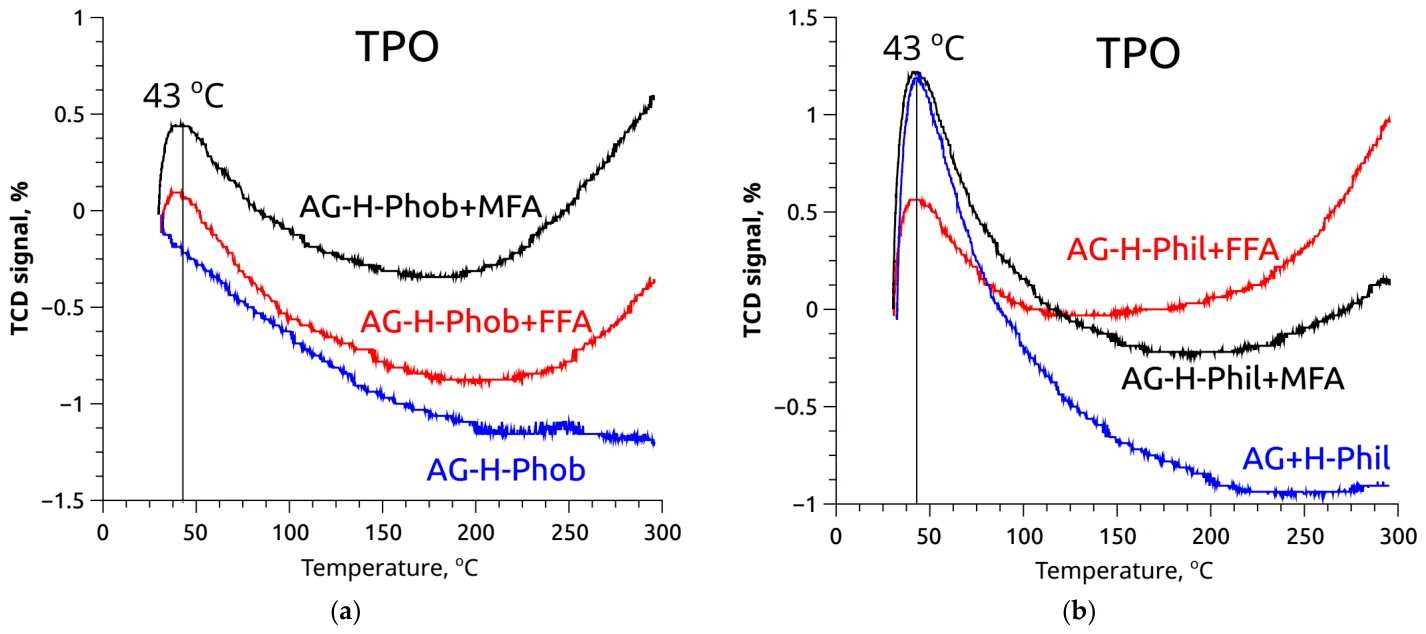
TPO data recorded over the temperature range from 30 °C to 300 °C for hydrophobic (**a**) and hydrophilic (**b**) aerogels (blue line) and the corresponding composite materials doped with MFA (black line) and FFA (red line).

To further distinguish these effects from contributions arising from residual volatile species, the TPO/TPD measurements were repeated after preliminary calcination of the samples in flowing He at 130 °C for 30 min. This treatment was intended to minimize the contribution of residual solvents responsible for the low-temperature features observed in the initial measurements (Figure 3).

**Figure 4:**
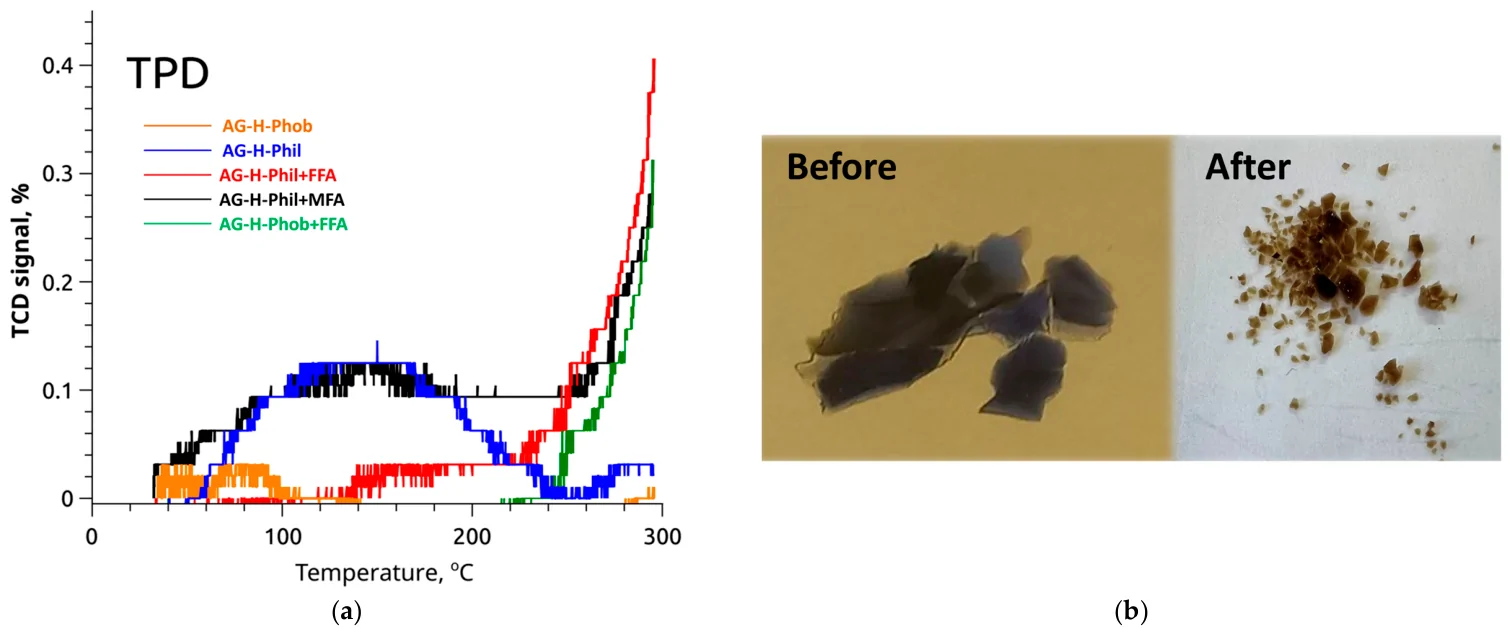
(**a**) TPD data recorded over the temperature range from 30 °C to 300 °C for hydrophobic (orange line) and hydrophilic (blue line) aerogels and the corresponding composite materials: AG-H-Phil+FFA (red line), AG-H-Phil+MFA (black line), and AG-H-Phob+FFA (green line). (**b**) Photographs of the AG-H-Phil+FFA sample before and after the sorption process.

The resulting TPO profiles show similar behavior in both pristine aerogels and fenamate-containing composites. In all samples, the dominant changes in the TCD response occur above approximately 200 °C, irrespective of the support’s hydrophilic or hydrophobic character and of the dopant’s identity. Calcination completely removes the low-temperature feature previously observed for AG-H-Phob, supporting its assignment to volatile species. The corresponding signal remains in the composites, although with substantially reduced intensity. In contrast, the low-temperature response of AG-H-Phil persists after calcination, most likely because the hydrophilic sample partially re-adsorbed moisture from ambient air during transfer between experiments. The TPD profiles provide an even more pronounced distinction. All fenamate-containing composites exhibit an intense desorption signal beginning at approximately 225 °C, whereas the undoped aerogels do not show a comparable TPD response within the investigated temperature range (see Figure 4). Consequently, the calcined samples confirm the previously observed tendency: the fenamate-containing composites retain CO_2_ more efficiently than the pristine aerogels under flow-through conditions. This behavior may be consistent with chemical interactions analogous to those previously identified for MFA and FFA in scCO_2_ [42,43].

At the same time, interpretation of the TPO data requires caution because the temperature ranges associated with oxidation of the aerogel framework and oxidation/decomposition of the fenamate dopants partially overlap. Therefore, an increase in the TPO response cannot be directly assigned to a particular chemical transformation of the dopant. Nevertheless, the TPD behavior is less readily explained by this overlap, as pristine aerogels do not exhibit a corresponding high-temperature CO_2_ desorption feature. An additional qualitative indication of chemical transformation is the color change observed in AG-H-Phil+FFA after CO_2_ sorption. A comparable color change was previously observed during formation of the FFA–CO_2_ reaction product [43,57]. Although the color change cannot independently establish the formation of a reaction product within the aerogel, its coincidence with the enhanced high-temperature CO_2_ retention provides additional support for this interpretation. The primary objective of this study is to demonstrate and qualitatively characterize this previously unobserved phenomenon. Quantitative assessment of the contributions of physisorption and chemisorption, as well as clarification of the molecular mechanism governing the interaction of CO_2_ with the composite, will require further experimental and computational investigations. These aspects will be addressed in future research.

Therefore, the evidence for a chemical contribution should be evaluated cumulatively rather than relying solely on the TPD signal. The current TPD results demonstrate the presence of an additional thermally persistent CO_2_ population in the fenamate-containing composites. Furthermore, previously reported spectroscopic and computational studies independently confirm the chemical reactivity of fenamates toward CO_2_. Collectively, these findings support the hypothesis that chemical interactions contribute to enhanced retention; however, they do not permit quantitative separation of this contribution from that of physical confinement or other specific interactions.

Hydrophilic and hydrophobic silica surfaces generate distinct interfaces for the confined fenamate phase, which may influence its interaction with carbon dioxide. However, the observed high-temperature carbon dioxide retention in composites derived from both types of matrices indicates that surface hydrophilicity or hydrophobicity alone does not determine this effect. Instead, surface chemistry likely modulates the outcome in combination with nanoconfinement and the specific interactions between fenamate and carbon dioxide.

### 2.2. BET Analysis

We employed the dynamic single-point BET method [58,59], which provides a rapid means of estimating changes in specific surface area at key experimental stages (see Table 1). Measurements were conducted sequentially within a single experimental cycle: initially on untreated samples, subsequently after preliminary thermal treatment at 130 °C under helium flow, and finally following the temperature-programmed CO_2_ sorption and desorption experiments. Figure S1 displays all experimental data used to estimate the specific surface area values.

This section analyses and compares the estimated specific surface area values of the composite materials under investigation. While specific surface area is not a direct measure of sorption capacity, the estimated values obtained in this experiment may reflect differences in the CO_2_ sorption characteristics of these systems. Therefore, these values should instead be regarded as indicative trends in the evolution of specific surface area. The pristine AG-H-Phil and AG-H-Phob samples exhibit high specific surface areas of 980 and 1018 m^2^ g^−1^, respectively. Following calcination, these values increase to 1016 and 1129 m^2^ g^−1^, which can be attributed to the removal of residual solvent and other volatile species from the pore network. The resulting values are typical for silica aerogels [60,61,62,63]. Fenamate loading decreases the apparent specific surface area, consistent with partial occupation or blockage of the pore space by the dopant molecules. The largest decrease is observed for AG-H-Phil+FFA, for which the specific surface area decreases to 449 m^2^ g^−1^, i.e., by more than a factor of two relative to the pristine AG-H-Phil matrix. Calcination increases the specific surface area of the composite materials by approximately 20% on average, consistent with the removal of residual volatile species and/or weakly retained surface material.

Importantly, the CO_2_ sorption/desorption cycle produces only minor changes in the specific surface area of the pristine aerogels, with deviations of no more than approximately 2%. Such changes are within the expected experimental uncertainty and indicate that exposure to CO_2_ under the present conditions does not cause substantial irreversible restructuring of the silica pore network. This observation is consistent with the TPD data, which show that pristine silica aerogels do not retain a significant amount of CO_2_ under flow-through conditions after the external CO_2_ supply is removed.

The behavior of the fenamate-containing composites is markedly different. After the CO_2_ sorption/desorption cycle, the FFA-containing materials show an increase in specific surface area of approximately 11% for AG-H-Phil+FFA and 5% for AG-H-Phob+FFA. These changes are unlikely to be explained simply by CO_2_-induced modification of the silica framework, since the corresponding pristine aerogels exhibit only negligible changes. Instead, they may reflect changes in the dopant’s state during interaction with CO_2_, including the formation and subsequent transformation of a reaction product. Such an interpretation is consistent with the previously reported FFA–CO_2_ reaction, in which the formation of an amorphous product was demonstrated spectroscopically [43].

In contrast, the changes observed in the MFA-containing composites are minimal, not exceeding approximately 1%. This difference between MFA- and FFA-containing systems may be related to the different stability of their CO_2_ reaction products. The product formed upon reaction of CO_2_ with MFA is highly unstable in air; its formation can therefore be detected during the TPO/TPD experiment while CO_2_ is continuously supplied, whereas after removal of the CO_2_ atmosphere it undergoes transformation toward the more stable polymorphic form II of MFA [8]. The comparatively small change in BET surface area after the sorption/desorption cycle is therefore consistent with the transient nature of the MFA–CO_2_ product and its subsequent transformation after CO_2_ removal.

Taken together, the TPO/TPD and BET results provide an internally consistent picture of the role of the fenamate dopants. The negligible change in surface area of the pristine aerogels after the CO_2_ cycle, combined with the absence of a pronounced high-temperature TPD signal, indicates that the silica matrix alone does not provide substantial long-term CO_2_ retention under flow-through conditions. In contrast, the emergence of high-temperature CO_2_ desorption features in the fenamate-containing composites demonstrates that the dopants introduce additional CO_2_-retention sites or states. Because the overall pore structure is not substantially altered by the CO_2_ cycle, this enhanced retention cannot be attributed solely to textural changes in the silica matrix [48].

Current data indicate that the increased retention of CO_2_ following sorption cannot be attributed solely to changes in the accessible textural characteristics of the silica matrix. Instead, this effect is more plausibly explained by a combination of pore confinement and interactions with the fenamate-containing phase. Previous studies demonstrating the chemical reactivity of MFA and FFA toward CO_2_ further suggest that a chemical contribution may account for the retained CO_2_. However, the present single-point BET and TPO/TPD measurements do not allow for quantification of the relative contributions of these mechanisms.

Importantly, the present results also reconcile the apparently different CO_2_ dynamics previously observed by NMR. Under continuous scCO_2_ exposure, the pristine silica aerogel can provide numerous accessible sites for CO_2_ localization and consequently exhibit relatively long correlation times. Once the external CO_2_ supply is removed, however, most of this physically localized CO_2_ is rapidly released. Fenamate incorporation introduces an additional population of more strongly retained CO_2_ species, as evidenced in the present experiments by the high-temperature TPD signal. Thus, the combination of NMR and TPO/TPD suggests that CO_2_ mobility and CO_2_ retention are not equivalent parameters: a material may exhibit extensive transient CO_2_ localization under continuous exposure yet fail to retain it after removal of the CO_2_ reservoir. Fenamate-containing composites, in contrast, appear to combine rapid CO_2_ uptake with a fraction of more strongly retained CO_2_ species.

Overall, these results support the view that the silica aerogel should not be considered merely as an inert porous support. Rather, confinement within the aerogel can modify the local environment and behavior of the fenamate dopant, thereby affecting its interaction with CO_2_ and the stability of the resulting products. The TPO/TPD and BET measurements therefore complement the previously reported NMR, IR, and computational results and provide evidence that the enhanced CO_2_ retention observed in the composite materials is governed by mechanisms extending beyond simple physical adsorption.

## 3. Experimental

The study examined composite materials consisting of H-Phil and H-Phob silica AG as carrier matrices, which were loaded with active pharmaceutical ingredients (APIs), specifically MFA and FFA [25]. Aerogel samples were synthesized at the D. I. Mendeleev Russian University of Chemical Technology (Moscow, Russia). Commercially available API samples from Sigma-Aldrich (Darmstadt, Germany) (CAS: 530-78-9; 61-68-7) were utilized without further purification. Six material types were analyzed: non-sorbed carrier matrices (AG-H-Phil and AG-H-Phob) and the corresponding fenamate-based composites (AG-H-Phil+MFA, AG-H-Phob+MFA, AG-H-Phil+FFA, and AG-H-Phob+FFA).

Gel samples were synthesized using sol–gel technology. Tetraethoxysilane (TEOS) served as the precursor, isopropyl alcohol (C_3_H_8_O) as the organic solvent, a 0.01 M aqueous solution of hydrochloric acid (HCl) as the acid catalyst, and a 0.06 M aqueous ammonia (NH_3_) solution as the cross-linking agent. For sol preparation, TEOS, C_3_H_8_O, and HCl were combined at a molar ratio of 1:7:3.5, respectively, and stirred for 24 h. An aqueous ammonia solution was then added to initiate gelation at a TEOS:NH_3_ molar ratio of 1:2.7, and the mixture was transferred into pre-prepared 4 mL molds. Gelation was completed within 30 min. The gels remained in the molds for 24 h to enhance structural integrity.

During the solvent exchange step, samples were immersed in isopropyl alcohol at a volume four times greater than the gel volume. This process was repeated three times at 24 h intervals to remove excess water and unreacted hydroxyl groups from the gel pores. For hydrophobization, the gels were placed in an alcoholic solution containing 10 wt.% of a silicone-based hydrophobizing agent (GKZh) and maintained at 60 °C for 8 h.

The final stage of aerogel preparation involved supercritical drying, which removed the solvent from the material pores while preserving the porous structure [64]. Carbon dioxide (CO_2_) served as the supercritical fluid. Supercritical drying was conducted at 120 bar, 40 °C, and a CO_2_ flow rate of 40 mL/min.

**Figure 5:**
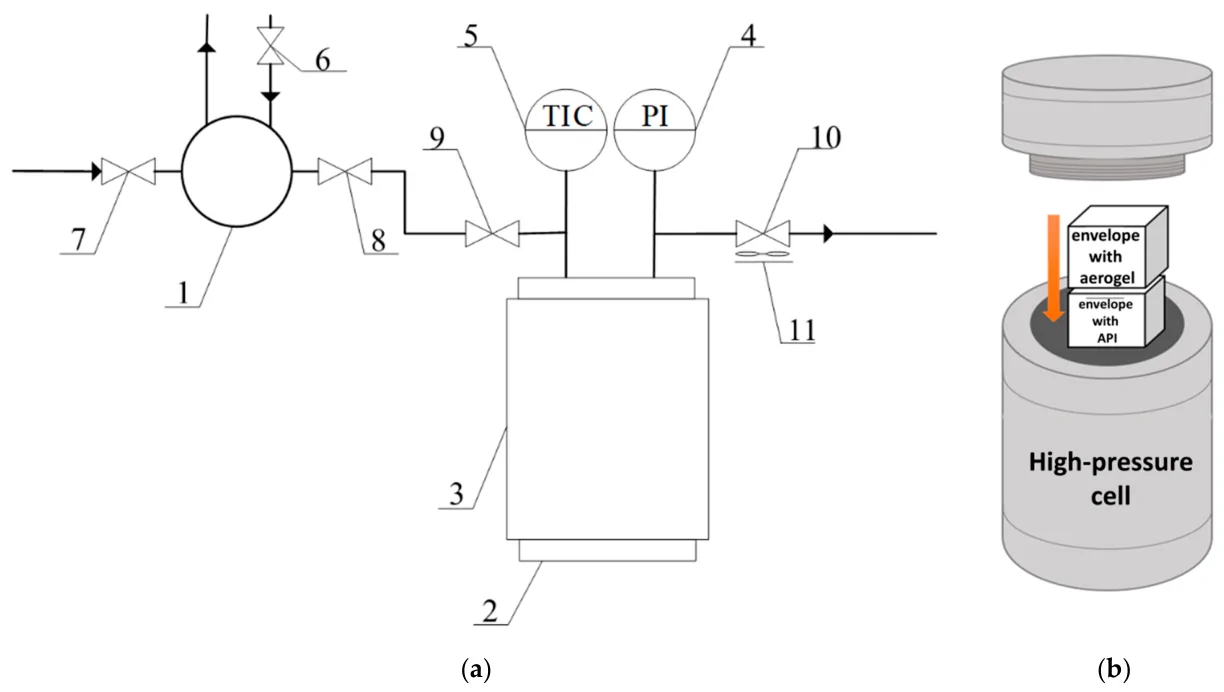
(**a**)—Schematic diagram of the supercritical adsorption setup: 1—liquid membrane pump; 2—high-pressure cell; 3—heating jacket; 4—pressure indicator (PI); 5—temperature indicating controller (TIC) with operator panel; 6—pump cooling shut-off valve; 7, 8—pump shut-off valves; 9—high-pressure cell inlet valve; 10—high-pressure cell outlet valve; 11—heating element. (**b**)—Schematic diagram illustrating the arrangement of envelopes containing the aerogel and API within the high-pressure cell.

Composite materials were additionally prepared using supercritical fluid technology. Supercritical CO_2_ has previously been demonstrated to be an effective medium for loading pharmaceutical compounds into silica aerogels, with drug loading depending on the properties of both the API and the aerogel matrix [18,21,24,65]. In particular, silica aerogels have been successfully impregnated with mefenamic acid using supercritical CO_2_, and the loading was shown to depend strongly on the concentration of MFA in the supercritical phase [66]. Process parameters of 200 bar and 70 °C were selected based on literature data regarding the solubility of mefenamic and flufenamic acids in supercritical CO_2_ [10]. Supercritical adsorption of the APIs into the aerogels was performed using the experimental setup illustrated in Figure 5. The use of supercritical CO_2_ for API loading is particularly advantageous because it enables drug incorporation into the porous matrix while minimizing the use of conventional organic solvents [65].

Aerogel (0.2 g) and API (0.2 g) samples were placed in separate filter-paper envelopes. The API envelope was positioned at the bottom of the high-pressure cell, with the aerogel envelope placed above it (see Figure 5b). After sealing the high-pressure cell, valve 10 was closed, and carbon dioxide pre-cooled to 5 °C was introduced into the 65 mL high-pressure cell (2) containing the envelopes. The liquid pump (1) was used to increase system pressure, while the heating jacket (3) maintained the target process temperature. Pressure and temperature were monitored using the pressure gauge (4) and temperature controller with operator panel (5). Upon reaching the desired pressure and temperature, valve 9 was closed to seal the cell. The supercritical adsorption process was conducted for 24 h. After completion, the pressure was released at a controlled rate of 5 bar per minute.

The sorption and desorption of CO_2_ were investigated using temperature-programmed reaction analysis. This method measures the difference in thermal conductivity between a reference carrier gas stream and the gas stream passing through the reactor containing the sample. Changes in the gas mixture composition due to oxidation, adsorption, or desorption alter the thermal conductivity, which is detected as a thermal conductivity detector (TCD) signal (Altamira Instruments, Twin Lakes, WI, USA). Under constant experimental conditions, the signal intensity is directly proportional to changes in the concentrations of the gaseous components. TPO and TPD experiments for the composite materials were conducted using an automated chemisorption analyzer (Altamira AMI-400TPx (Altamira Instruments, Twin Lakes, WI, USA)). For TPO experiments, samples of AG and the composite materials were placed in a U-shaped quartz tube and heated to the specified temperature at a rate of 10 °C per minute in a flow of 10% CO_2_ in an inert helium atmosphere (flow rate: 30 mL/min).

TPD measurements were carried out under a flow of pure helium, with the heating rate and gas flow rate kept constant. Temperature-programmed desorption is widely used to characterize the interactions between adsorbed species and porous surfaces, as well as to assess the strength and distribution of adsorption sites [67,68].

We determined the specific surface area of the samples using the BET method under dynamic flow conditions on an AMI-400 automated analyzer (Altamira Instruments, Twin Lakes, WI, USA). Measurements employed a 30% N_2_/He mixture at a relative pressure (P/P_0_) of 0.3. This relative pressure was chosen as a representative condition for rapid single-point estimation of the BET surface area of the predominantly mesoporous aerogel materials [69].

We employed the single-point approach primarily to monitor relative changes in specific surface area during sequential experimental treatments, rather than to achieve comprehensive quantitative textural characterization. The principal advantage in this study was that surface area estimates could be obtained directly within the experimental sequence, enabling observation of changes associated with gas–solid interactions and subsequent treatments without removing the sample from the reactor. Therefore, the reported values should be regarded as comparative estimates of specific surface area and interpreted primarily in terms of their relative changes and trends.

## 4. Conclusions

The experimental results demonstrate that incorporation of MFA and FFA, collectively referred to as fenamates, into silica aerogels leads to increased CO_2_ retention following sorption under flow-through conditions. In contrast to plain hydrophilic and hydrophobic silica aerogels, fenamate-containing composites exhibit a distinct high-temperature CO_2_ desorption property, indicating the formation of CO_2_-retaining states not characteristic of the original silica matrices.

Results indicate that the increased CO_2_ retention cannot be explained by accessible area or by the hydrophilic or hydrophobic character of the silica matrix. Persistence of the high-temperature TPD peak after initial thermal treatment further suggests that the effect is not attributable to residual volatile substances. Additionally, single-point BET measurements provide further evidence that the observed retention does not result from significant irreversible alterations in the accessible surface area of the original aerogel matrices.

Together with previous NMR, spectroscopic, and computational data, these findings support a model that considers the combined effects of spatial confinement, matrix-fenamate interactions, and specific interactions between fenamates and CO_2_. A reversible chemical transformation of the fenamate-containing phase under confinement may explain the increased retention, consistent with previously reported reactivity of MFA and FFA toward CO_2_. However, the TPO/TPD measurements in this study are insufficient to quantify the relative contributions of physisorption, confinement, and chemisorption, or to identify a specific reaction product definitively.

The observed differences between MFA-containing and FFA-containing composites underscore the significance of the molecular dopant and its local chemical environment. Overall, these results highlight the potential of fenamate-functionalized silica aerogels as composite materials with tunable post-sorption CO_2_ retention properties and identify nanoconfinement as a key factor influencing interactions between the confined fenamate phase and CO_2_.

## Figures and Tables

**Table 1:** Estimated specific surface area values for the investigated samples determined using the single-point BET method.

Aerogel/Composite Sample	Initial Sample, m^2^/g	After Calcination, m^2^/g	After CO_2_ Sorption/Desorption, m^2^/g
AG-H-Phil+FFA	449	574	634
AG-H-Phil+MFA	833	981	974
AG-H-Phob+MFA	1007	1060	1068
AG-H-Phob+FFA	688	784	823
AG-H-Phil	980	1016	1022
AG-H-Phob	1018	1129	1107

## Data Availability

The original contributions presented in this study are included in the article and Supplementary Materials. Further inquiries can be directed to the corresponding author.

## Supplementary Materials

The following supporting information can be downloaded at https://www.mdpi.com/article/10.3390/molecules31183248/s1. Figure S1: The N_2_ adsorption–desorption isotherms of AG-H-Phil (a), AG-H-Phob (b), AG-H-Phil+MFA (c), AG-H-Phob+MFA (d), AG-H-Phil+FFA (e), and AG-H-Phob+FFA (f), used for BET surface area analysis.
