# Supplementary material for: Nanoconfinement-Enhanced CO2 Retention in Fenamate-Loaded Silica Aerogels
Source: Molecules. 2026 Sep 14;31(18):3248. doi: 10.3390/molecules31183248 (PMC13609461; doi:10.3390/molecules31183248)
Supplement: Supplementary file 1 [file molecules-31-03248-s001.zip › molecules-4553648-supplementary.pdf]

Supporting information

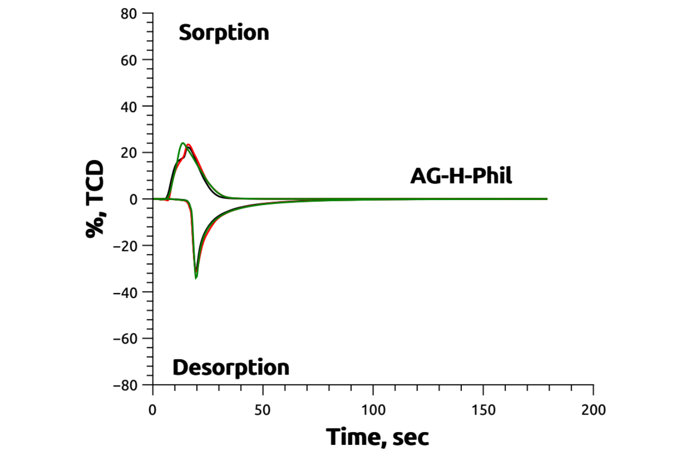

a

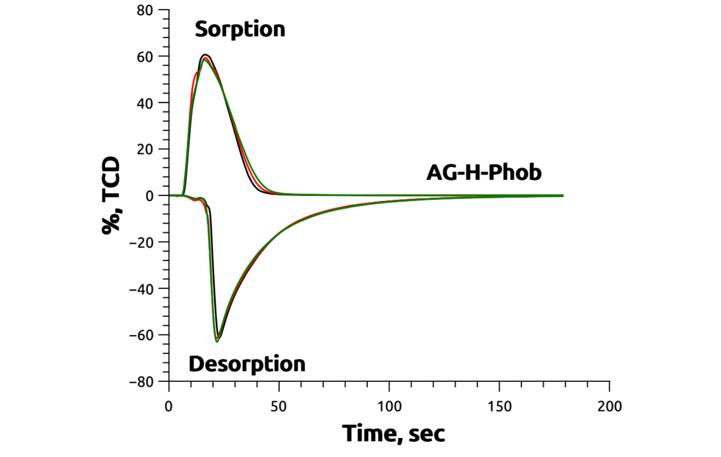

b

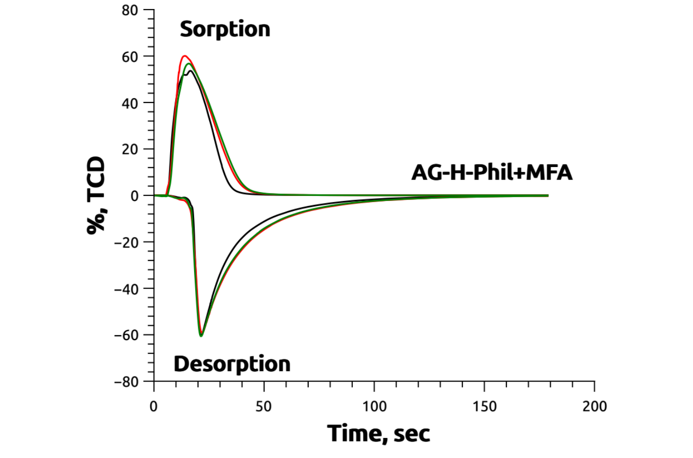

c

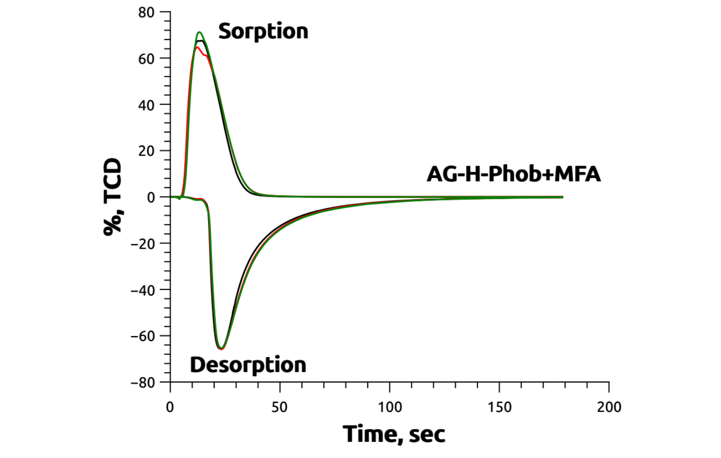

d

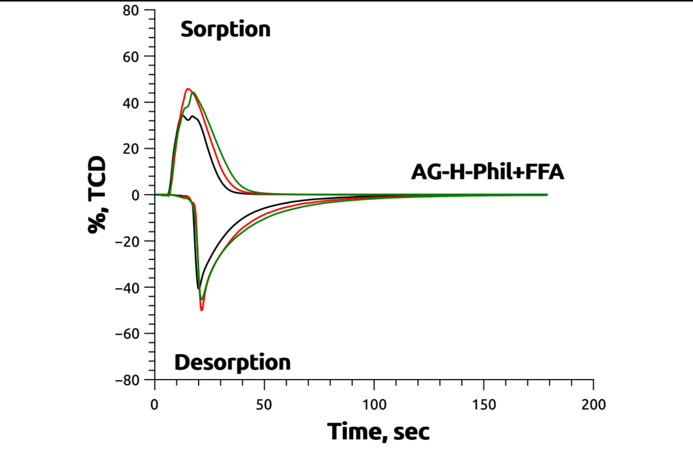

e

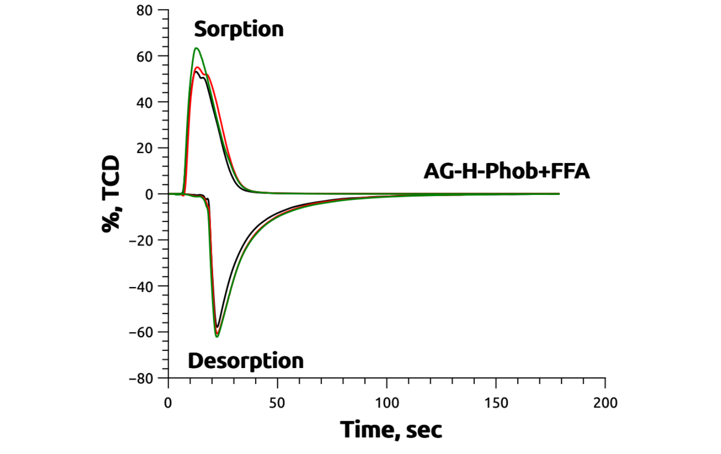

f

Figure S1. The N_2 adsorption–desorption isotherms of AG-H-Phil (a), AG-H-Phob (b), AG-H-Phil+MFA (c), AG-H-Phob+MFA (d), AG-H-Phil+FFA (e), and AG-H-Phob+FFA (f), used for BET surface area analysis.
